# Network Pharmacology and Bioinformatics Study of Geniposide Regulating Oxidative Stress in Colorectal Cancer

**DOI:** 10.3390/ijms242015222

**Published:** 2023-10-16

**Authors:** Yingzi Wu, Jinhai Luo, Baojun Xu

**Affiliations:** Guangdong Provincial Key Laboratory IRADS, Department of Life Sciences, BNU-HKBU United International College, Zhuhai 519087, China

**Keywords:** oxidative stress, CRC, network pharmacology, molecular docking, bioinformatics, geniposide

## Abstract

This study aims to identify the mechanism of geniposide regulating oxidative stress in colorectal cancer (CRC) through network pharmacology and bioinformatics analysis. Targets of geniposide, oxidative stress-related targets and targets related to CRC were applied from databases. The hub genes for geniposide regulating oxidative stress in CRC were identified with the protein–protein interaction (PPI) network. Furthermore, we applied Gene ontology (GO) and Kyoto Encyclopedia of Genes and Genomes (KEGG) enrichment to analyze the hub genes from a macro perspective. We verified the hub genes by molecular docking, GEPIA, HPA and starBase database. We identified five hub genes: *IL1B, GSK3B, NOS3, RELA and CDK4*. GO analysis results suggested that the anti-colorectal cancer effect of geniposide by regulating oxidative stress is possibly related to the influence of multiple biological processes, including response to temperature stimulus, response to alkaloid, nitric oxide biosynthetic process, nitric oxide metabolic process, reactive nitrogen species metabolic process, cellular response to peptide, etc. KEGG enrichment analysis results indicated that the PI3K–Akt signaling pathway, IL-17 signaling pathway, p53 signaling pathway, NF-κB signaling pathway and NOD-like receptor signaling pathway are likely to be the significant pathways. Molecular docking results showed that the geniposide had a good binding activity with the hub genes. This study demonstrates that geniposide can regulate oxidative stress in CRC, and induction of oxidative stress is one of the possible mechanisms of anti-recurrence and metastasis effects of geniposide against CRC.

## 1. Introduction

Colorectal cancer is one of the top three malignancies with the highest mortality rate today [1]. Clinical treatments for colorectal cancer mainly include endoscopy, surgical resection, radiotherapy, immunotherapy and palliative chemotherapy targeted therapy, extensive surgery, and local ablation of metastases, which can extend the survival of patients to a certain extent [1]. However, due to the occurrence of tumor drug resistance, recurrence and metastasis, colorectal cancer is still a difficult problem in medical treatment [2]. It is urgent to explore effective and low-toxicity therapeutic methods to combat colorectal cancer [3].

Fructus Gardeniae (FG) is the dried fruit of *Gardenia jasminoides* Ellis (GjE), belonging to the family Rubiaceae [4]. Geniposide (C_17_H_24_O_10_), a well-known iridoid glycoside compound, is one of the main bioactive components of FG [5]. Previous studies have shown that geniposide has many positive effects, such as anti-inflammatory, anti-tumor, blood glucose and lipid regulation, antibacterial, anti-depression, anti-oxidation, immune regulation, and so on [6]. Geniposide has also been shown to greatly reduce the growth of various kinds of cancer cell lines, including diffuse large B-cell lymphoma cells [7], medulloblastoma cells [8] and gastric MKN45 cells [9]. Though many emerging benefits of geniposide have been found, the studies on geniposide anti-colorectal cancer are still rare.

Oxidative stress is an imbalance between the production of reactive oxygen species (ROS) and the body’s antioxidant defenses. This imbalance contributes to chronic inflammation, which can lead to precancerous conditions. ROS can influence cellular functions, including gene regulation and apoptosis, making oxidative stress a significant factor in cancer development [10,11,12]. Network pharmacology is an emerging, systematic approach to exploring the complex mechanisms of traditional Chinese medicine (TCM). It deviates from the traditional ‘one drug, one target’ model, highlighting that multiple substances can interact with various genes or proteins. This approach allows for a more holistic understanding of medicine and disease interactions, providing insights into multiple medications and targets [13,14]. In order to determine the utility of geniposide as a medicine, we decided to explore its impact on CRC using a network pharmacological method.

**Figure 1:**
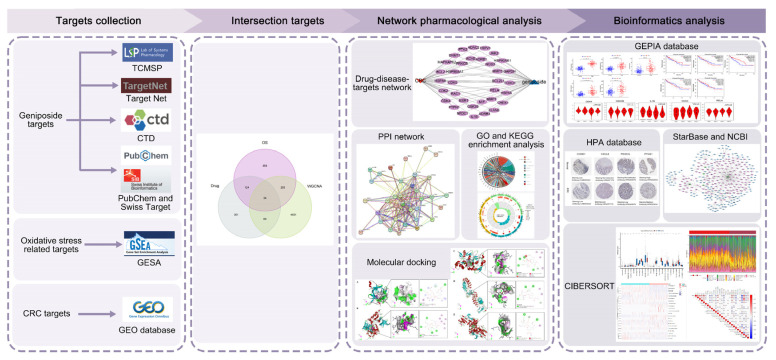
A flowchart of this study on geniposide regulating oxidative stress in colorectal cancer.

In this study, network pharmacology was used to identify the pharmacologic target network of geniposide, and bioinformatics was used to compile information on the relevant targets of colorectal cancer. The intersection targets were subjected to GO and KEGG enrichment analysis. To clarify the pathophysiology of colorectal cancer and offer a fresh perspective on clinical intervention therapy, we verified the hub genes by using molecular docking, GEPIA, HPA, and the starBase database. The study flowchart is shown in Figure 1.

## 2. Results

### 2.1. Construction of Target Network Map of Geniposide

**Figure 2:**
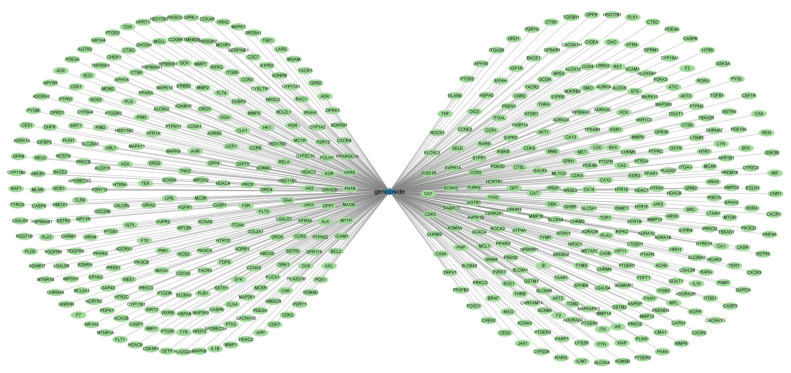
Geniposide-targets network, 548 geniposide-related targets in total were obtained.

We retrieved 102, 473, 24 and 7 geniposide-related targets, respectively, through the Swiss Target Prediction, TargetNet, CTD (Comparative Toxicogenomics Database) and TCMSP databases (Traditional Chinese Medicine Systems Pharmacology Database). After merging and deduplicating the related targets of the four databases, 548 geniposide-related targets in total were obtained. Subsequently, we used the Cytoscape software to construct a network diagram of geniposide and its related targets, as shown in Figure 2.

### 2.2. Screening of Gene Modules and Construction of Co-Expression Network

**Figure 3:**
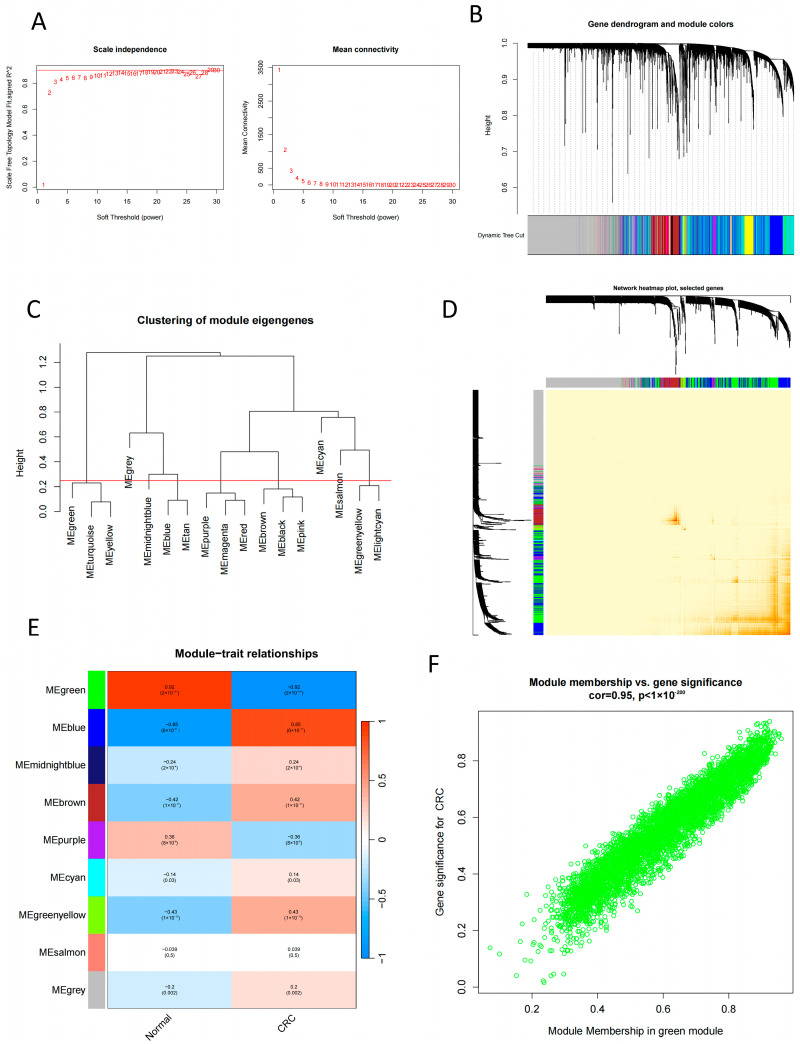
WGCNA analysis chart; (**A**) Network topology for different soft-thresholding powers. Numbers in the plots indicate the corresponding soft thresholding powers. (**B**) Gene dendrogram obtained by clustering the dissimilarity based on consensus Topological Overlap with the corresponding module colors indicated by the color row. (**C**) Dendrogram of consensus module eigengenes obtained by WGCNA on the consensus correlation. The red line is the merging threshold, and groups of eigengenes below the threshold represent modules whose expressions profiles should be merged due to their similarity. (**D**) The heatmap depicts the TOM among all modules included in the analysis. The progressively darker red color represents an increasing overlap. (**E**) Module–trait associations. Red represents positive correlation, while blue represents negative correlation. (**F**) Module membership of green module, which represents a positive correlation of green module genes in different CRC samples.

To identify the hub gene modules associated with CRC, we used the WGCNA algorithm to establish co-expression networks and modules in healthy people and CRC patients. We calculated the variance of the expression of each gene in GSE44076 and then selected the top 25% of genes with the most significant variance for further analysis. Co-expressed gene modules were identified when the soft power value was set to 10, and the scale-free R2 equaled 0.9, as illustrated in Figure 3A. Using the dynamic cutting algorithm, a total of nine co-expression modules with different colors were obtained when presenting the heat map of the topological overlap matrix (TOM), as shown in Figure 3B–D. Subsequently, these genes in the nine color modules were sequentially applied to analyze the similarity and adjacency of the co-expression of the module-clinical features (normal group and CRC group). Furthermore, the green module showed the most substantial relationship with CRC, which included 5157 genes, as shown in Figure 3E. Finally, we observed a positive correlation of green module genes in different CRC samples, as indicated in Figure 3F.

### 2.3. Screening of Core Genes

**Figure 4:**
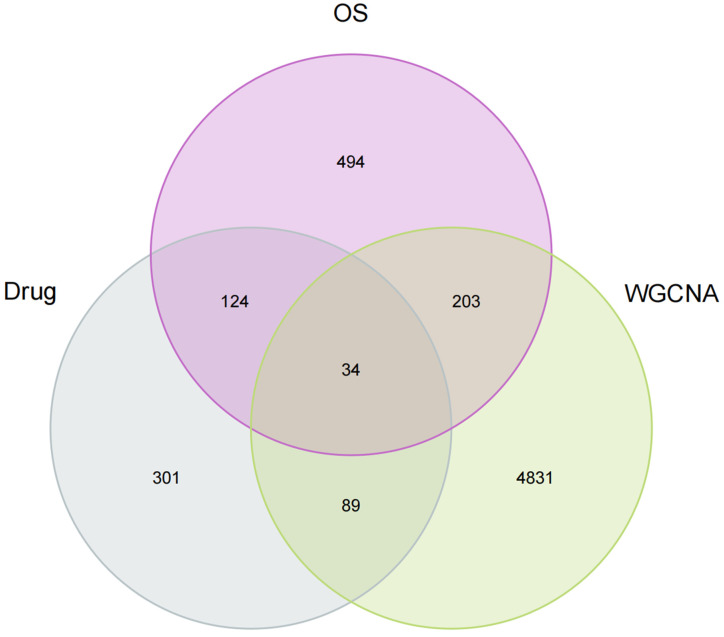
The intersecting targets between potential targets of geniposide, oxidative stress-related genes, and CRC-related genes.

**Figure 5:**
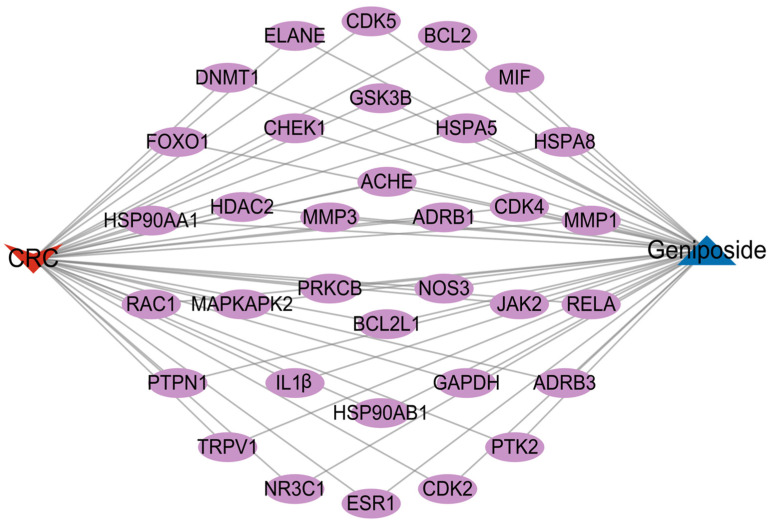
Compound–disease–targets network, purple represents the targets related to compound as well as disease.

In order to explore the role of geniposide regulating oxidative stress against colorectal cancer, we intersected the targets of geniposide with the most critical module genes and oxidative stress-related genes in CRC, and obtained a total of 34 core genes as shown in Figure 4, including *DNMT1, HSP90AA1, PTPN1, ESR1, PTK2, RELA, CDK4, CDK5, ADRB1, MAPKAPK2, TRPV1, CDK2, NOS3, JAK2, MIF, GSK3B, ACHE, MMP3, NR3C1, HSP90AB1, ADRB3, MMP1, BCL2, ELANE, HDAC2, RAC1, PRKCB, BCL2L1, GAPDH, HSPA8, HSPA5, CHEK1, FOXO1, IL1B*. Then, we constructed a compound–disease–targets network diagram using the Cytoscape software, as illustrated in Figure 5.

### 2.4. Core Genes PPI Network Construction and Enrichment Analysis

**Figure 6:**
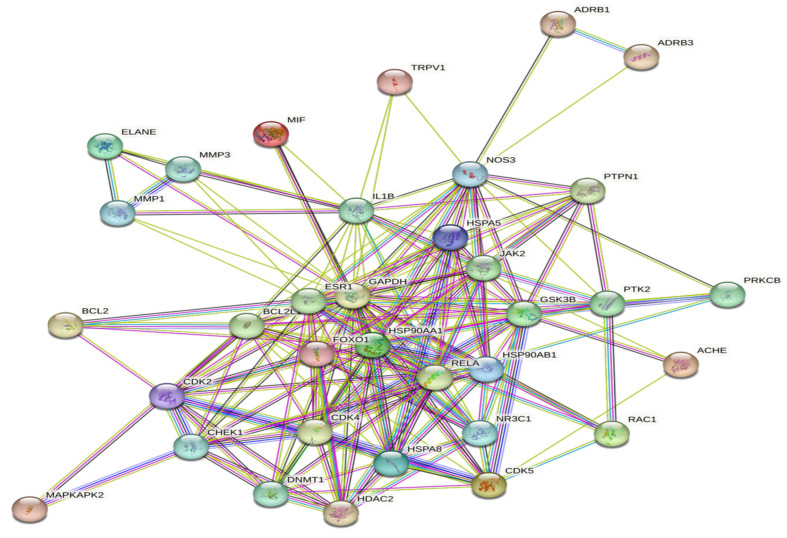
Protein–protein interaction Network.

We imported the above 34 core genes into the String database to obtain the PPI network between the core genes, as shown in Figure 6.

**Figure 7:**
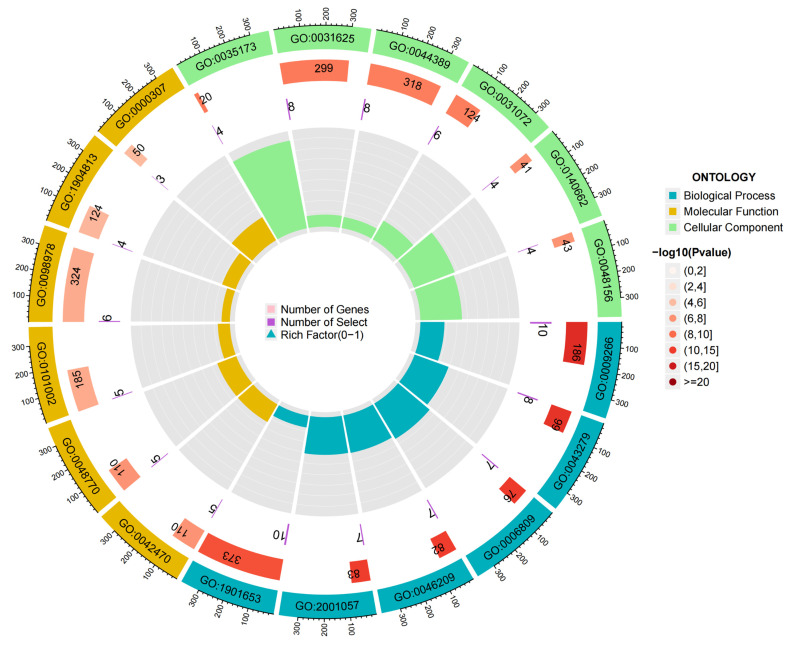
GO enrichment analysis; blue represents Biological Process (BP), yellow represents Molecular Function (MF), and green represents Cellular Component (CC).

Through the “cluster profiler” package of the R language software, the GO function enrichment analysis of the 34 core genes of geniposide anti-colorectal cancer by regulating oxidative stress was carried out. A total of 1582 GO entries were obtained (*p*-value < 0.05), and the main category was the biological process (BP), cell composition (CC), and molecular function (MF), as illustrated in Table 1. Among them were 1395 BP entries, mainly involving response to temperature stimulus, response to alkaloid and nitric oxide biosynthetic process, and so on. There were 61 CC entries, mainly related to melanosome, pigment granule, ficolin-1-rich granule, and so on. Also,126 MF entries were mainly related to histone kinase activity, ubiquitin protein ligase binding, ubiquitin-like protein ligase binding, and so on. The above analysis showed that geniposide is likely to act on these cell structures, affect these biological processes, and exert these molecular functions to achieve the purpose of anti-colorectal cancer by regulating oxidative stress, as illustrated in Figure 7.

**Figure 8:**
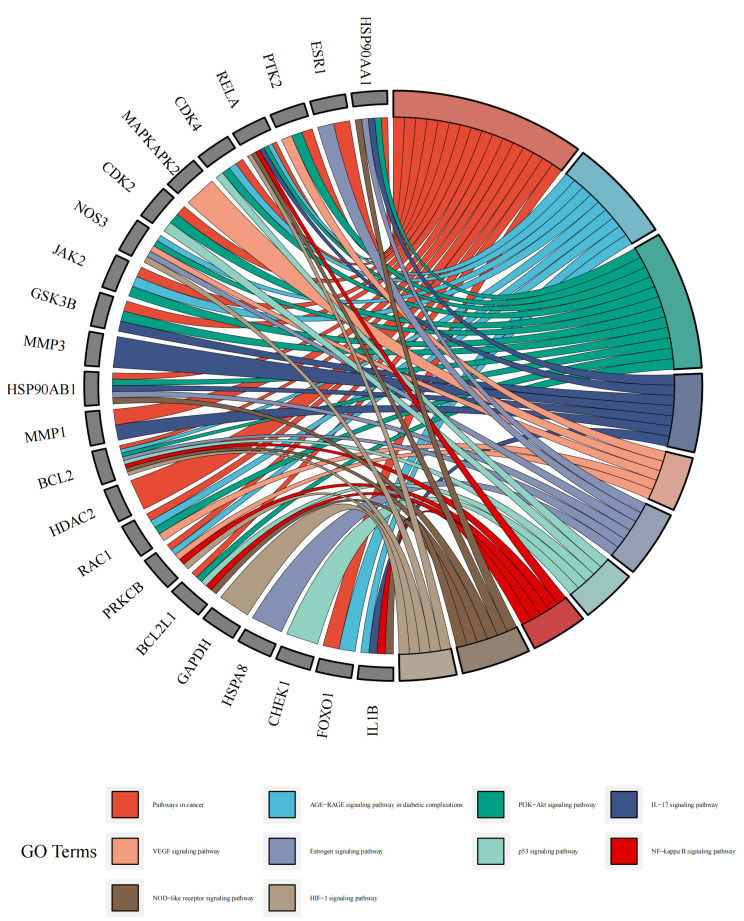
KEGG enrichment analysis; red represents signaling pathway in cancer, dark blue represents IL-17 signaling pathway, light green represents p53 signaling pathway, red represents NF-κB signaling pathway, and brown represents NOD-like receptor signaling pathway.

By utilizing the “cluster profiler” package of the R language software, the KEGG pathway enrichment analysis of the 34 core genes of geniposide on CRC was carried out. The results showed 87 signal pathways (*p*-value < 0.05) in which geniposide interfered with CRC, mainly involving pathways in the cancer signaling pathway, PI3K–Akt signaling pathway, IL-17 signaling pathway, p53 signaling pathway, NF-κB signaling pathway, and NOD-like receptor signaling pathway, as shown in Figure 8. 

**Figure 9:**
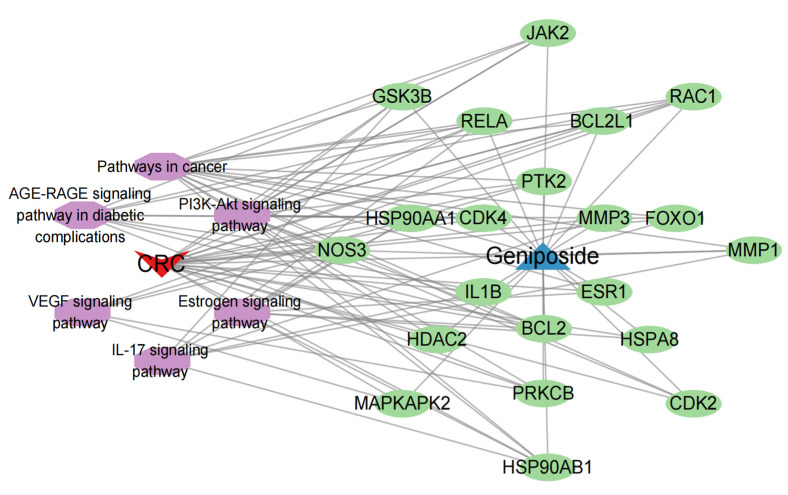
Compound–disease–therapeutic targets-core pathways network; red triangles represent diseases, blue triangles represent compounds, purple ovals represent pathways, and green ovals represent targets.

Finally, we imported the compound–disease–therapeutic targets-core pathways network file into Cytoscape software to draw the pathway network diagram, which displayed more intuitively the multi-component-multi-target action characteristics of active ingredients of traditional Chinese medicine in the treatment of CRC as illustrated in Figure 9.

### 2.5. Hub Genes Screening and Correlation Analysis of Pyroptosis-Related Genes

**Figure 10:**
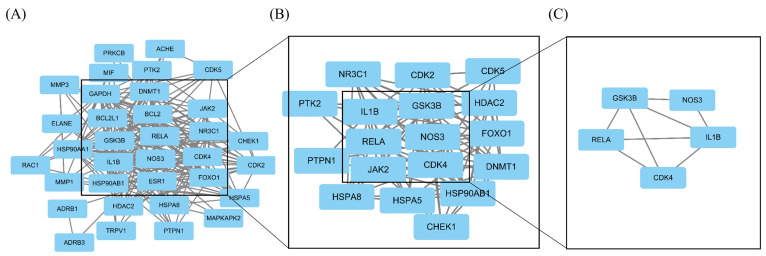
Hub genes screening map; (**A**) represents interaction relationship of 34 core genes; (**B**) represents interaction relationship of genes whose degrees are higher than the average degree; (**C**) represents interaction relationship of hub genes.

**Figure 11:**
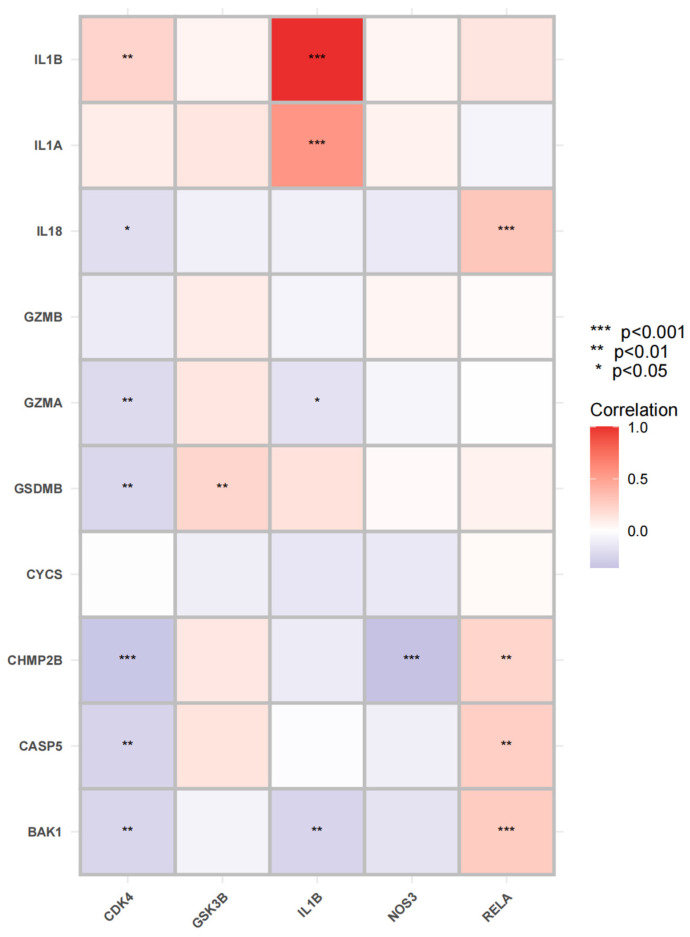
Correlation analysis between hub genes and pyroptosis hotspot genes; the horizontal coordinate represents hub genes, and the vertical coordinate represents hotspot genes of pyroptosis.

As shown in Figure 10, we screened 34 core genes and found that when the degree value was greater than the average, 18 genes were selected (*IL1B, GSK3B, NOS3, RELA, CDK4, HSP90AB1, NR3C1, FOXO1, JAK2, CDK2, HSPA8, CDK5, HSPA5, CHEK1, HDAC2, PTK2, DNMT1, PTPN1*). As shown in Figure 10B- C, according to degree, all of the top five genes (*IL1B, GSK3B, NOS3, RELA, and CDK4*) are above 15, which were selected as hub genes for subsequent analysis. The functional enrichment of GO and KEGG suggested that the mechanism of geniposide’s anti-colorectal cancer regulating oxidative stress may be related to cell pyroptosis, and oxidative stress is one of the vital induction methods of cell pyroptosis. In order to explore the correlation between hub genes and pyroptotic genes, we conducted a correlation analysis between hub genes and pyroptotic hotspot genes *(IL1A, IL1B, IL18, GZMB, GZMA, GSDMB, CYCS, CHMP2B, CASP5, BAK1*). The results showed that *1L1B, CDK4* and *RELA* are closely related to pyroptosis hotspot genes, as shown in Figure 11.

### 2.6. Hub Genes ceRNA Network Construction

**Figure 12:**
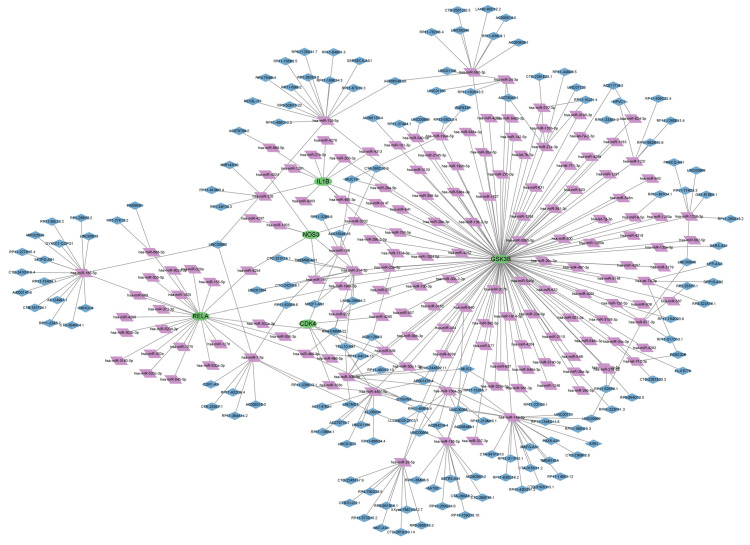
ceRNA network diagram of hub genes; purple represents miRNA, blue represents lncRNA, and green represents mRNA.

We constructed a ceRNA network based on hub genes with starBase, the miRDB database and the miRnada database. The network includes 319 nodes (5 hub genes, 157 miRNAs and 157 lncRNAs) (Figure 12). In detail, a total of 119 lncRNAs could competitively bind miRNA hsa-miR-4318, hsa-miR-3147, and hsa-miR-29a-5p to regulate *GSK3B*. In the ceRNA network, 11 lncRNAs can combine with miRNA hsa-miR-495-3p, hsa-miR-326, and hsa-miR-4303 to regulate the hub gene *IL1B*. miRNA hsa-miR-4318, hsa-miR-4280, hsa-miR-103b, hsa-miR-506-3p, hsa-miR-198, hsa-miR-568, hsa-miR-149-3p, and hsa-miR- 486-5p bind to regulate the hub gene *CDK4*. miRNA hsa-miR-4318, hsa-miR-4254, hsa-miR-761, hsa-miR-4297, hsa-miR-3202, hsa-miR-1303, hsa-miR-4324, and hsa-miR-214-3p bind to regulate the hub gene *NOS*3. In addition, 18 lncRNAs could combine with has-miR-156-5p, hsa-miR-858-3p and hsa-miR-7-5p to regulate the hub gene *RELA*.

### 2.7. PPI Network Analysis, Differential Expression Lever, Pathological Staging, and OS Analysis of Hub Genes

**Figure 13:**
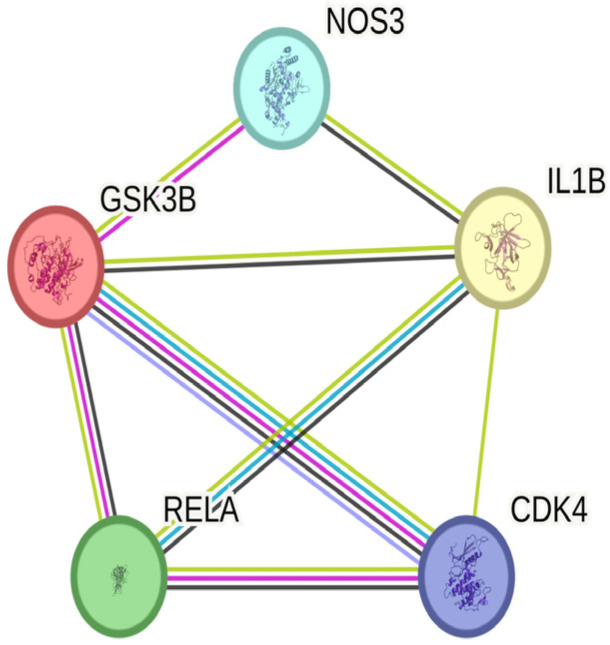
Hub genes PPI network.

**Figure 14:**
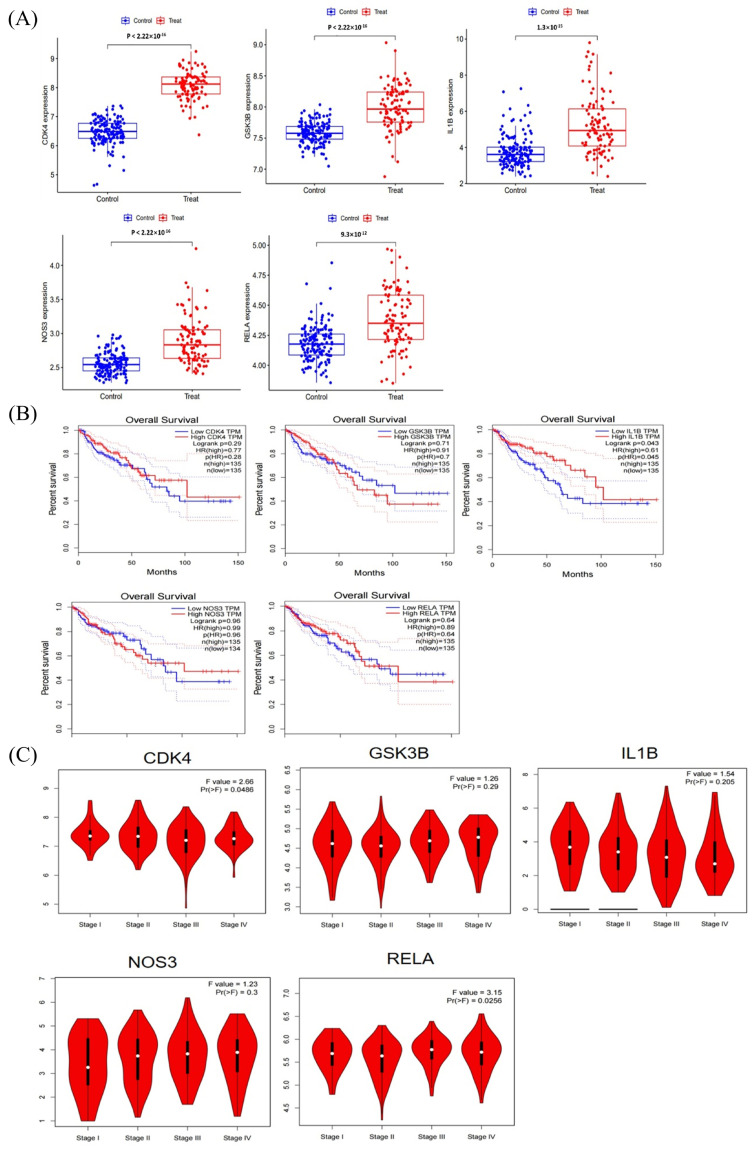
mRNA expression level, pathological stage, and OS in the GEPIA database. (**A**) Box plots showing the mRNA expression levels of *CDK4, GSK3B, IL1B, NOS3*, and *RELA*. Red represents tumor group, while blue represents normal group. (**B**) The line charts show the overall survival (OS) of hub genes in GEPIA. The survival curve compares the patients with high (red) and low (blue) expression in CRC. (**C**) The violin diagram indicates the stage plot of mRNA expression level and pathological stage in the GEPIA database.

**Figure 15:**
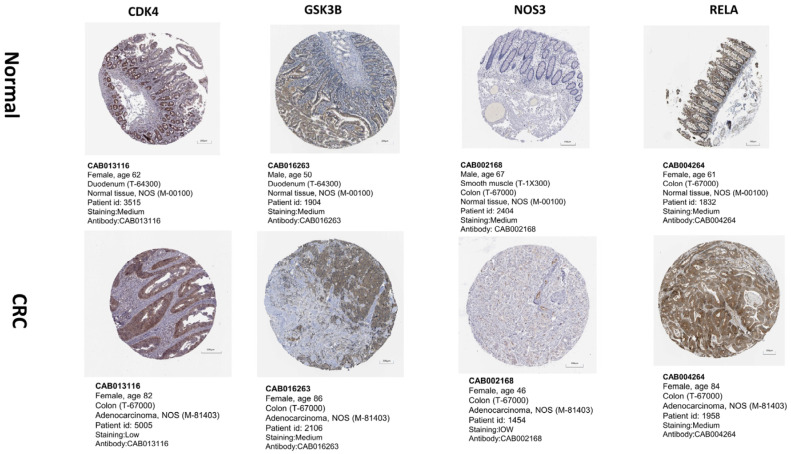
The protein expression levels of hub genes with microscope’s magnification of 200 μm in the HPA database.

To understand the interrelationships among the hub genes, we conducted a PPI network analysis on them, as shown in Figure 13. Subsequently, we conducted differential expression levels, pathological stage, and OS in the GEPIA database. The results showed that the expression of hub genes in the CRC group was more remarkable than that in the normal group with *p*-value < 0.05, as shown in Figure 14A. Moreover, the prognosis of the *IL1B* high expression group was statistically significant, as illustrated in Figure 14B. Pathological staging showed that as the disease progressed, the mRNA levels of *IL1B* gradually decreased while the mRNA levels of *NOS3* gradually increased, as shown in Figure 14C. The results of the HPA database showed that the other hub genes were expressed to varying degrees in colorectal cancer tissue, except for *IL1B*, which had no corresponding data in the database (Figure 15).

### 2.8. Immune Infiltration Analysis of Hub Genes

**Figure 16:**
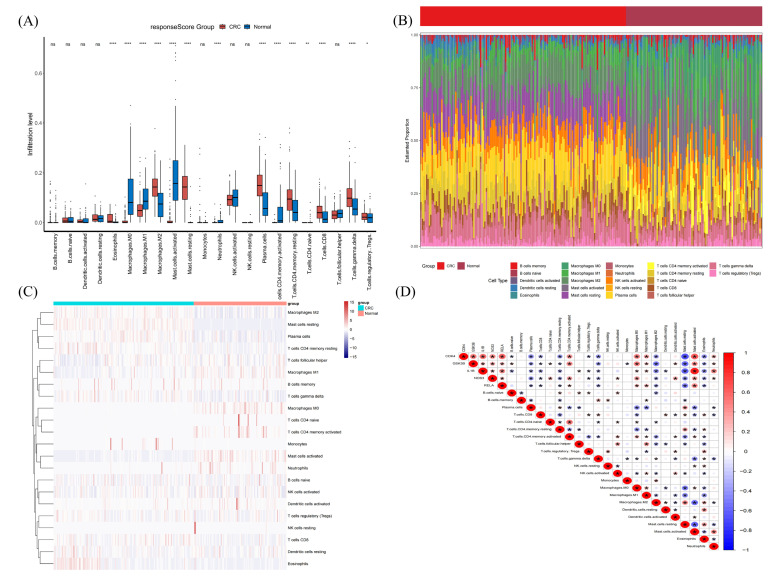
Immune infiltration analysis of hub genes; (**A**) reflects the difference in immune cell infiltration between normal group and cancer group; (**B**) shows the difference in immune cell infiltration between each sample; (**C**) represents the difference in immune cell infiltration between the normal group and the tumor group, it was statistically analyzed, and “ns” indicated that the difference was statistically insignificant; (**D**) indicates that five genes were strongly correlated with each other, with red representing positive correlation, blue representing negative correlation, and “*” indicating statistically significant correlation, and “*” means *p*-value < 0.05, “**” means *p*-value < 0.01, “****” means *p*-value < 0.001.

The difference in immune microenvironment between the normal group and the CRC group was analyzed by CYBERSORT. The difference in immune cell content between the normal group and the CRC group can be visually seen in Figure 16A,B. Statistical “wilcox.test” method was used to compare the difference between the two groups (Figure 16C). Eosinophil, Macrophages M0, Macrophages M1, Macrophages M2, Mast cells activated, Mast cells resting, Neutrophils, Plasma cells, T cells CD4 memory activated, T cells CD4 memory resting, T cells CD4 naive, T cells CD8, T cells gamma delta, T cells regulatory (Tregs) and so on were significantly different (*p*-value < 0.05) between the two groups. Among them, Eosinophil, Macrophages M2, Plasma cells, T cells CD4 memory resting, T cells CD8, T cells gamma delta, T cells regulatory (Tregs) were the most invasive immune cells in the CRC group. The M1/M2 ratio imbalance had significance in the formation of malignancies, immune escape, and subsequent metastatic treatment resistance. The relationship between the hub genes and immune cells is shown in Figure 16D, which suggested that there was a strong positive correlation between the hub genes that were all strongly positively correlated with Macrophages M0, Macrophages M1, and Mast cells activated, while a strong negative correlation with Plasma cells, T cells CD8, T cells CD4 memory resting, T cells gamma delta, Macrophages M2, Mast cells resting, and Eosinophils.

### 2.9. Molecular Docking Results

**Figure 17:**
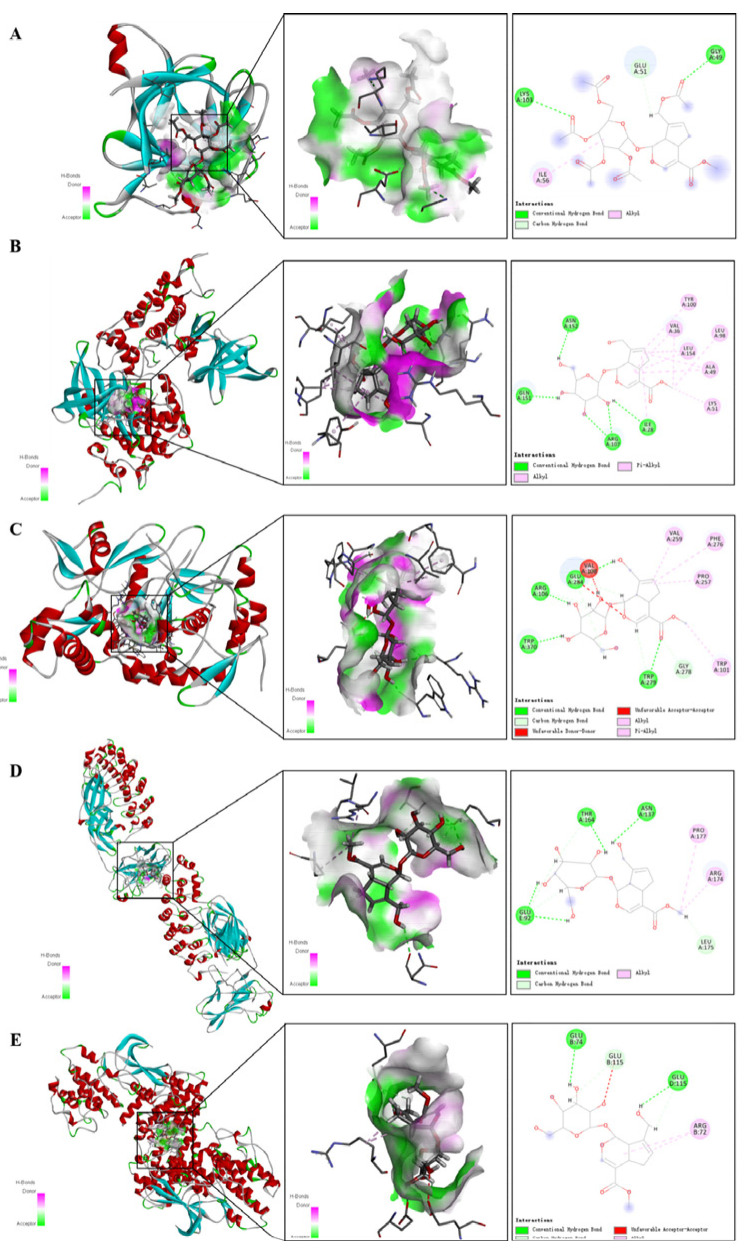
Schematic diagram of docking between geniposide and *IL1B, GSK3B, NOS3, RELA* and *CDK4* (**A**–**E**). (**A**) Represents the molecular binding of geniposide with *IL1B*; (**B**) represents the molecular binding of geniposide with *GSK3B*. (**C**) Represents the molecular binding of geniposide with *NOS3*; (**D**) represents the molecular binding of geniposide with *RELA*; (**E**) represents the molecular binding of geniposide with *CDK4*.

Results of *IL1B*_1I1B: The binding energy between the small molecule and the receptor was −6.2kcal/mol, which demonstrated that it had a good interaction force and formed hydrogen bond associations mainly with GLY A49 and LYS A103, as shown in Figure 17A. Results of *GSK3B*_1J1B: The binding energy between the small molecule and the receptor was −7.5 kcal/mol, which demonstrated that it had a good binding effect. Small molecules interacted with receptor proteins, mainly forming hydrogen bonds with ASN A152, GLNA151, ARGA107, ILEA28, and LYS A103, as shown in Figure 17B. Results of *NOS3*_3E7S: The binding energy between the small molecule and the receptor was −7.0 kcal/mol, which indicated that it has a good binding effect. Small molecules interacted with receptor proteins, mainly with GLUA284, ARGA106, TRPA370, and TRPA279, and obvious hydrogen bond interactions were found, as shown in Figure 17C. Results of *RELA*_1NFI: The binding energy of the small molecule to the receptor was −6.3 kcal/mol, which indicated that it had a good binding effect. Small molecules interacted with receptor proteins mainly through the formation of hydrogen bonds and hydrophobic bond forces, including hydrogen bonds THRA164, ASNA131, and GLUE92, as shown in Figure 17D. Results of *CDK4*_3G33: The binding energy of the small molecule to the receptor was −6.4 kcal/mol, which indicated that it had a good binding effect. Small molecules interacted with receptor proteins, including hydrogen bonds formed by GLUB74 and GLUD175, as shown in Figure 17E. The docking scores of geniposide and hub genes can be seen in Table 2.

## 3. Discussion

The prevalence of colorectal cancer has been rising annually in recent years because of changes in human living habits, and the age at which it occurs is getting younger. In the past 10 years, more and more natural products have been confirmed to have anti-tumor effects. Geniposide is the primary active component in the herbal remedy Gardenia jasminoides used in traditional Chinese medicine. It has activities in anti-inflammatory, antimicrobial, anti-tumor, anti-oxidation, and immunological regulatory, according to preliminary research. Oxidative stress is produced when the ratio of reactive oxygen species (ROS) production to antioxidant effectiveness is out of balance. Excessive ROS synthesis can cause cellular damage and is one of the factors resulting in many disorders, including cancer. This study aims to explore the molecular mechanism of geniposide in the treatment of colorectal cancer by regulating oxidative stress and the bioinformatics analysis of its hub genes to provide a reference for the development of new treatment strategies. Through the Swiss Target Prediction, TargetNet, CTD and TCMSP databases, we collected 548 targets of geniposide. Then, we searched the CRC dataset in the GEO database and used the WGCNA algorithm to establish the co-expression network and modules of healthy people and CRC patients and a collection of 5157 genes with positive correlations among different CRC samples was identified. Since the occurrence of oxidative stress is closely related to the occurrence and development of malignant tumors, we need to explore the potential mechanism of whether geniposide can treat CRC by regulating oxidative stress. We employed the GSEA website to collect 855 oxidative stress-related genes and took the intersection of the three to obtain the hub genes of geniposide treatment on CRC.

In addition, GO enrichment analysis showed that the biological functions mainly involved positive regulation of response to temperature stimulus, response to alkaloid, nitric oxide biosynthetic process, nitric oxide metabolic process, reactive nitrogen species metabolic process, cellular response to peptide, and so on. From the enrichment results of GO, the synthesis and metabolism of nitric oxide and the metabolic process of reactive nitrogen were significantly enriched. Nitric oxide (NO) is synthesized by many cell types involved in immunity and inflammation. It also controls the functional activity, growth, and death of a wide range of immunological and inflammatory cell types, including Macrophages, T lymphocytes, antigen-presenting cells, Mast cells, Neutrophils, and natural killer cells [15]. Inflammation and cancer are related in numerous ways, including proliferation, invasion, angiogenesis, and metastasis [16]. Furthermore, inflammation-mediated cytokine release regulated by diverse cells within the tumor microenvironment plays a significant role in these processes [17], which reveals that the progression of inflammation can, to some extent, trigger tumor growth. Cell components mainly included melanosome, pigment granule, ficolin-1-rich granule, glutamatergic synapse, ficolin-1-rich granule lumen, cyclin-dependent protein kinase holoenzyme complex, and so on. Molecular functions mainly included histone kinase activity, ubiquitin protein ligase binding, ubiquitin-like protein ligase binding, heat shock protein binding, ATP-dependent protein folding chaperone, tau protein binding and other related gene targets. 

KEGG pathway enrichment analysis showed that there were 87 signaling pathways (*p*-value < 0.05) in which geniposide-regulated oxidative stress intervened in CRC, mainly involving pathways in the cancer signaling pathway, PI3K–Akt signaling pathway, IL-17 signaling pathway, p53 signaling pathway, NF-κB signaling pathway and NOD-like receptor signaling pathway. Despite the fact that the PI3K–AKT signaling pathway governs various cellular processes such as differentiation, metabolism, survival, and apoptosis, its abnormal activation contributes to the development of colorectal cancer malignancy [18]. IL-17 is a very flexible pro-inflammatory cytokine that is required for a range of functions, such as host defense, tissue repair, inflammatory disease pathogenesis, and cancer progression [19]. Previous research has revealed that IL-17 activates a variety of intracellular signaling pathways, including NF-κB and MAPKs such as p38, JNK, and ERK1/2, all of which are involved in the regulation of tumor incidence and progression. The tumor suppressor gene TP53 is one of the most frequently altered genes in numerous malignancies, including colorectal cancer [20]. According to some studies, the p53 signaling pathway can decrease cancers via modulating cell cycle arrest [21], angiogenesis inhibition [22], DNA repair and angiogenesis [23], apoptosis [22], etc. As a result, altering the p53 signaling pathway may be a therapeutic approach for colorectal cancer treatment. The NF-κB signaling pathway controls cell proliferation, apoptosis, angiogenesis, inflammation, metastasis, and treatment resistance in CRC. When this route is activated, it causes the production of proliferation-related genes, including cyclin D1, cyclin E, and cyclin-dependent kinase (CDK)-2, as well as IL-6 and Myc. Because abnormal NF-κB regulation is frequently observed in tumor cells, inhibiting this cascade may decrease cell proliferation [24]. One of the main routes involved in the regulation of pyroptosis is the NOD-like receptor signaling pathway, and NLRP3 is the most studied NLR in recent years. NLRP3 recruits ASC and caspase-1 during activation, which is required for the cleavage and maturation of the inflammatory cytokines IL-1B and IL-18, as well as the subsequent inflammatory cell death known as pyroptosis [25]. Li Liang et al. reported that oxymatrine has an anti-cancer effect via LRPPRC inhibition, mitophagy induction, and NLRP3 inflammasome suppression [26]. Qin et al. found that Atractylide I has anti-colorectal cancer properties via suppressing Drp1-mediated mitochondrial fission and NLRP3 inflammasome activation in colitis-associated colorectal cancer [27]. Therefore, modulating the NOD-like receptor signaling pathway could be one possibility for anti-colorectal cancer therapy. These signaling pathways may collaborate in the molecular mechanism of geniposide-regulated oxidative stress response against colorectal cancer.

Pyroptosis is intimately related to oxidative stress and has recently become a research hotspot. The primary signaling pathways enriched by KEGG were involved in regulating the occurrence and progression of pyroptosis. Therefore, we also conducted a study on the correlation between hub genes and hotspot pyrogenic genes, the result of which suggested that hub genes were closely related to pyroptosis hotspot genes, especially *IL1B, CDK4* and *RELA*. The activation of pyroptosis promotes the cleavage of the pro-inflammatory cytokines IL-1 and IL-18 into active and secretory forms [25]. The ceRNA network of hub genes further suggested that there was a strong positive correlation between the five hub genes, which were all strongly positively correlated with Macrophages M0, Macrophages M0, Macrophages M1, and Mast cells activated, while a negative correlation with Plasma cells, T cells CD8, T cells CD4 memory resting, T cells gamma delta, Macrophages M2, Mast cells resting, and Eosinophils. As an important cell component in the tumor microenvironment, Macrophages play an important role in the composition and functional composition of the tumor microenvironment. M2 Macrophages in the microenvironment surrounding the tumor express a high level of IL10, which limits the tumor microenvironment’s immune response. Moreover, Plasma cells and T CD8 are crucial immune cells that assist the immune system in removing antigens, viruses, and tumors, as well as suppress the incidence and proliferation of cancer cells [28].

Our PPI network analysis results showed that *IL1B, GSK3B, NOS3, RELA* and *CDK4* were related to oxidative stress induced by geniposide against colorectal cancer. We also performed a bioinformatics analysis of these hub genes. Analysis of *GSK3B* shows the dual activity of inhibiting or promoting tumors [29]. Considering that *GSK3B* suppresses the Wnt signaling pathway, it is presumed to be an inhibitor of tumors in general [30]. In contrast, current research suggests that *GSK3B* can activate the NF-κB signaling cascade by increasing NF-κB transcriptional activity in the nucleus, hence promoting malignancy [31]. *RELA*(P65) is a transcription factor in the nuclear factor κB (NF-κB) family [32,33]. NF-κB activation promotes CRC by speeding up cell proliferation and angiogenesis, inhibiting apoptosis, and promoting cell invasion and metastasis, and NF-κB is involved in all stages of CRC development [33]. *CDK4* is one of the cell cycle regulators that controls the G1 to S phase of the cell cycle. Because multiple cancer treatments may reduce *CDK4* expression, this protein has been regarded as a critical target protein in a variety of malignancies [34]. Several investigations have shown that *CDK4* is overexpressed in cancer [35,36] and inhibiting *CDK4* overexpression improves clinical treatment efficacy for breast cancer, melanoma, liposarcoma, and mantle cell lymphoma [37], which suggests that CDK4 could be a therapeutic target for cancer [38]. *NOS3* is known to be involved in NO synthesis, primarily in endothelial cells, and is linked to cardiovascular disorders such as hypertension, atherosclerosis, and diabetes mellitus [39]. It has been observed that *NOS3* inhibits apoptosis and promotes angiogenesis, proliferation, invasiveness, and immunosuppression in malignant tumors [40]. Furthermore, *NOS3* levels were found to be inversely associated with progression-free survival and overall survival (OS) in patients with metastatic colorectal cancer [41].

Hub gene *IL-1B* is produced and secreted by a range of tumor cell types, including immune cells, fibroblasts, and cancer cells. It has also been connected to a wide range of physiological activities. It has the capacity to control cytokine production and gene expression as well as cellular adhesion and migration, angiogenesis, and immune response [42]. Additionally, *IL-1B* often encourages the growth and invasion of cancer cells as well as neo-angiogenesis and immune cells that infiltrate tumors [43]. Furthermore, a mouse model of adenomatous polyposis coli (APC) colon cancer showed significant levels of *IL-1B* and *IL-1A* [44]. Furthermore, it has recently been demonstrated that IL-1R1 deficit in Neutrophils promotes bacterial invasion and tumor aggressiveness, while IL-1R1 deficiency in epithelial cells decreases tumorigenesis in an APC model [45]. Inhibiting IL-1B, however, may or may not be helpful for patients based on its polymorphism, depending on the cancer kind or stage, the primary type of immune cells present in the tumor microenvironment, and the anti-cancer treatment utilized [45]. Therefore, before considering *IL1B* as a potential method to treat colorectal cancer, it is important to further examine the correlations between *IL1B* polymorphism and prognosis. In summary, in this study, we employed four databases—Swiss Target Prediction, Target Net, CTD, and TCMSP—to identify genes and targets associated with geniposide. Each of these databases has its unique strengths and coverage, and their combined use ensures a more comprehensive and accurate target identification. GO and KEGG enrichment analyses provided useful insights into the biological processes and signaling pathways these genes may be involved in. Notably, enrichment results in cancer signaling pathways and the PI3K–Akt signaling pathway may suggest potential mechanisms of geniposide’s anti-CRC effects. Molecular docking results indicated possible interaction mechanisms between geniposide and its primary targets, providing direction for subsequent in vitro and in vivo experiments. However, these docking results are predictive and will require experimental validation through techniques such as X-ray crystallography or NMR in the future. Our data showed a significant increase in certain immune cells, such as M2 Macrophages and Tregs, in the CRC group. These cells are generally associated with immune suppression and cancer progression. These results suggest that geniposide may exert its anti-cancer effects by modulating the immune response in the tumor microenvironment. Despite the promising findings, our study has several limitations that should be acknowledged. First, the target identification relies heavily on in silico databases, which might not encompass all possible interactions or biological activities of geniposide. Second, our work is predominantly computational and lacks experimental validation. Molecular docking results are purely predictive and should be confirmed through in vitro and in vivo experiments. Third, while our study points to alterations in immune cell populations within the CRC tumor microenvironment, the mechanistic relationship between geniposide and immune modulation remains unclear and warrants further investigation. Lastly, our analyses were based on publicly available datasets, the quality and comprehensiveness of which we could not control. Future work should aim to address these limitations, perhaps by employing a wider variety of databases, conducting laboratory experiments for validation, and exploring the impact of geniposide on immune cells through more targeted assays.

Geniposide displayed high binding activity with the hub genes, as demonstrated in molecular docking results. We discovered that the mRNA levels of five hub genes were significantly expressed in CRC tissues, and the prognostic value of ILIB was significantly different (*p*-value < 0.05). With the evolution of pathological stages (Stage I to Stage IV), the mRNA expression of *IL1B* gradually decreased, while the mRNA expression of *NOS3* gradually increased. The above analysis results were basically consistent with literature reports. 

## 4. Materials and Methods

### 4.1. Prediction of Related Targets of Geniposide

We utilized PubChem (https://pubchem.ncbi.nlm.nih.gov/, accessed on 2 July 2023) to search for geniposide-related SMILES numbers and used Swiss Target Prediction databases (http://www.swisstargetprediction.ch/, accessed on 2 July 2023) and TargetNet database (http://targetnet.scbdd.com/home/index/, accessed on 2 July 2023) [46] with the default page setting to predict their corresponding targets. Then, we used the CTD database (https://ctdbase.org/, accessed on 2 July 2023) [47] and the TCMSP database (https://old.tcmsp-e.com/tcmsp.php, accessed on 2 July 2023) [48] with “geniposide” as a keyword to obtain geniposide-related targets. Finally, the UniProt database (http://www.uniprot.org/, accessed on 2 July 2023) [49] was used to correct the entered protein target name to its Official Symbol and set the species as “human”.

### 4.2. Colorectal Cancer Data Collection and Weighted Gene Co-Expression Network Analysis (WGCNA)

We took “Colorectal cancer” as keywords in Gene Expression Omnibus (GEO, http://www.ncbi.nlms.nih.gov/geo, accessed on 3 July 2023) [50] to retrieve the CRC dataset. As a training dataset for discovery, the GSE44076 dataset is constructed from the GPL13667 [HG-U219] Affymetrix Human Genome U219 Array platform and contains colon tissues from 149 CRC patients and 99 healthy people. All data used in the study were obtained from GEO. Therefore, neither ethical approval nor informed consent was required. We applied the R package of “WGCNA” (version 1.72-1, accessed on 3 July 2023), which was performed to determine the co-expression module. We applied only the top 25% of genes with the largest difference in subsequent WGCNA analyses to ensure the accuracy of the quality results. We chose an optimal soft power to construct a weighted adjacency matrix and further transformed it into a topological overlap matrix (TOM). The module was obtained using the TOM similarity and difference metric (1-TOM) based on the hierarchical clustering tree algorithm when the minimum module size was set to 100. Each module was assigned to a random color. Module eigengene represented the global gene expression profile for each module. Relations between modules and disease states were represented by modular significance (MS). Correlations between a gene and its clinical phenotype were described with genetic significance (GS).

### 4.3. Collection of Oxidative Stress Genes and Acquisition of Core Genes

To explore whether the potential mechanism of geniposide in the treatment of CRC is related to oxidative stress, we collected 855 oxidative stress- related genes from previous literature [51]. Then, we took the three intersections to get the core genes of geniposide in the treatment of CRC by using the “VennDiagram” package (version 1.7.3, accessed on 4 July 2023) in R software (version 4.32.12, accessed on 4 July 2023). The core genes were visually networked with the help of Cytoscape software (version 3.10.0, accessed on 4 July 2023).

### 4.4. Construction and Enrichment Analysis of Protein-Protein Interaction (PPI) Networks in Core Genes

The above-screened core genes were imported into the String database (https://cn.string-db.org/, version 11.5, accessed on 5 July 2023). The protein–protein interaction network (PPI) was constructed with a minimum required interaction score ≥ 0.4 as the screening condition. The PPI network diagram and tsv file were downloaded. To further explore the biological functions of core genes, GO gene enrichment analysis and KEGG signaling pathway analysis were performed and visualized on core genes by using the “clusterProfiler” (version3.17, accessed on 5 July 2023), “org.Hs.eg.db” (version 2.10, accessed on 5 July 2023), and “ggplot2” (version 3.4.3, accessed on 5 July 2023) packages in R software to explore the main molecular biological processes and signaling pathways of potential targets of geniposide. Finally, the compound–disease–therapeutic targets-core pathways network diagram was constructed using Cytoscape software (version 3.10.0, accessed on 5 July 2023) with nodes in the network representing compound, disease, targets, and pathways. Nodes were connected by edges that represented different meanings depending on the ways the network was built.

### 4.5. Screening of Hub Genes and Correlation Analysis of Genes Related to Pyroptosis

After 34 core genes were imported into Cytoscape software, the degree value was calculated, and hub genes were selected according to their degree value. To further understand the potential role of geniposide in the treatment of hub genes against CRC, we analyzed the correlations between hub genes and hotspot genes of pyroptosis. First, we collected 44 pyroptosis-related genes from previous study [52] Subsequently, the pyroptosis-related genes in CRC samples were screened by using the “limma” package (version 3.56.2, accessed on 5 July 2023) in R software with |logFC| > 0.585, and *p*-value < 0.05 as the screening conditions, and 10 related pyroptosis hotspot genes in CRC were screened (CASP5, CHMP2B, CYCS, GZMB, IL18, GZMA, BAK1, GSDMB, IL1A, IL1B). Finally, a correlation analysis was performed on hub genes and hotspot pyroptosis-related genes.

### 4.6. Construction of Hub Genes ceRNA Network

StarBase database (http://starbase.sysu.edu.cn, accessed on 5 July 2023), miRDB database (http://www.mirdb.org/, accessed on 5 July 2023) and miRanda database (http://www.microrna.org/, accessed on 5 July 2023 [53]) were used to predict mRNA–miRNA interaction pairs of hub genes, respectively. The predicted miRNAs were finally searched in the spongeScan database (http://spongescan.rc.ufl.edu, accessed on 5 July 2023) and miRNA–lncRNAs were screened to obtain the ceRNA network of mRNA–miRNA–lncRNAs. Among them, the Cytoscape software was used for visual analysis.

### 4.7. PPI Network Analysis, mRNA Expression Lever Analysis, Overall Survival (OS) Analysis, Pathological Staging and Immunohistochemical Analysis of Hub Genes

We imported the hub genes screened above into the String database (https://string-db.org/, accessed on 7 July 2023) and used “minimum required interaction score ≥ 0.4” as the screening condition to construct a PPI network, download and save the PPI network diagram and tsv format file. We used the “limma” package (version 3.56.2, accessed on 5 July 2023) in R software to analyze the differential expression of hub genes in CRC. Then, the hub genes were input into the online tool GEPIA (http://gepia.cancer-pku.cn/index.html, accessed on 7 July 2023) [54] to verify their pathology Staging and overall survival (OS) in TCGA-COAD. The protein expression of core genes was studied in the HPA database (https://www.proteinatlas.org/, accessed on 7 July 2023) [55].

### 4.8. Immune Cell Infiltration Analysis

We utilized the CIBERSORT (http://cibersortx.stanford.edu, accessed on 8 July 2023) [56] algorithm to determine the relative proportions of 22 invading immune cell types in each tissue. The immunological score of each sample was calculated using the “ESTIMATE” algorithm (version 2.0.0, accessed on 8 July 2023). In addition, the associations of signature genes with the number of infiltrating immune cells were determined by using the “Spearman” (version 1.11.3, accessed on 8 July 2023) rank correlation analysis in R software. The graphing method of the “ggplot2” package (version 3.4.0, accessed on 8 July 2023) was used to visualize the resulting correlations.

### 4.9. Molecular Docking

Geniposide was imported into Chem3D software (version 14.0.0.17, accessed 10 July 2023) to construct its chemical structure formula and minimize its energy, then saved as a PDB format file. The hub genes were brought into the PDB database (https://www.rcsb.org/, accessed on 10 July 2023), corresponding proteins were downloaded as PDB format files and imported into PyMol software (version 2.5.5, accessed on 10 July 2023) to remove water molecules and small molecules. The protein was imported into the autodocktool software (version 4.2.6, accessed on 10 July 2023) for hydroprocessing and saved as a PDBQT format file. Finally, PyMol and MOE software (version 2.1, accessed on 10 July 2023) were used for molecular docking and visualization analysis [57].

## 5. Conclusions

In this research, we successfully determined the molecular pathways of geniposide-regulated oxidative stress anti-colorectal cancer therapy. This study also demonstrated that hub genes (*IL1B, GSK3B, NOS3, RELA, and CDK4*) were most likely to be involved in the effects of oxidative stress brought on by geniposide in the prevention of colorectal cancer. The underlying mechanisms through which geniposide curbs colorectal cancer effects were the regulation of the multiple BP, including response to temperature stimulus, response to alkaloid, nitric oxide biosynthetic process, nitric oxide metabolic process, reactive nitrogen species metabolic process, cellular response to peptide, and so on. We identified six critical pathways (pathways in cancer signaling pathway, PI3K–Akt signaling pathway, IL-17 signaling pathway, p53 signaling pathway, NF-κB signaling pathway, and NOD-like receptor signaling pathway) involved in the treatment of colorectal cancer with geniposide by regulating oxidative stress. 

Hence, our findings showed a synergistic effect between various anti-colorectal cancer core targets, numerous molecular pathways, and geniposide treatment for colorectal cancer. The findings of molecular docking revealed that geniposide had a regulatory effect on the anti-colorectal cancer core targets. Further bioinformatic analysis showed the impact of hub genes on disease prognosis and other aspects. Furthermore, the results from molecular docking, bioinformatics, and network pharmacology screening were all consistent, proving the validity of network pharmacology in this study. These results, therefore, provide a basis for the future development of geniposide-based anti-colorectal cancer medications.

## Figures and Tables

**Table 1:** GO enrichment results.

Classification	Serial Number	Biological Function
BP	GO:0009266	response to temperature stimulus
BP	GO:0043279	response to alkaloid
BP	GO:0006809	nitric oxide biosynthetic process
BP	GO:0046209	nitric oxide metabolic process
BP	GO:2001057	reactive nitrogen species metabolic process
BP	GO:1901653	cellular response to peptide
CC	GO:0042470	melanosome
CC	GO:0048770	pigment granule
CC	GO:0101002	ficolin-1-rich granule
CC	GO:0098978	glutamatergic synapse
CC	GO:1904813	ficolin-1-rich granule lumen
CC	GO:0000307	cyclin-dependent protein kinase holoenzyme complex
MF	GO:0035173	histone kinase activity
MF	GO:0031625	ubiquitin protein ligase binding
MF	GO:0044389	ubiquitin-like protein ligase binding
MF	GO:0031072	heat shock protein binding
MF	GO:0140662	ATP-dependent protein folding chaperone
MF	GO:0048156	tau protein binding

**Table 2:** Docking scores of geniposide and hub genes.

Compound	PDB ID	Gene	Binding Energy
geniposide	1I1B	*IL1B*	−6.2
geniposide	1J1B	*GSK3B*	−7.5
geniposide	3E7S	*NOS3*	−7.0
geniposide	1NFI	*RELA*	−6.3
geniposide	3G33	*CDK4*	−6.4

## Data Availability

The data, analytic methods, and study materials that support the findings of this study are available from the corresponding author upon reasonable request.

## Abbreviations

BP	Biological process
CC	Cellular component
CDK4	Cyclin-dependent kinase 4
CRC	Colorectal cancer
DC	Degree centrality
DL	Drug-likeness
GO	Gene Ontology
GSK3B	Glycogen Synthase Kinase 3 Beta
IL1B	Interleukin lB
KEGG	Kyoto Encyclopedia of Genes and Genomes
MF	Molecular function
NOS3	Nitric oxide synthase
OB	Oral bioavailability
OS	Overall survival
PDB	Protein Data Bank
PPI	Protein–protein Interaction
ROS	Active oxygen species
RNS	Reactive nitrogen species
TCM	Traditional Chinese Medicine
TCMSP	Traditional Chinese Medicine Systems Pharmacology
